# Identification of adult Philadelphia-like acute lymphoblastic leukemia using a FISH‐based algorithm distinguishes prognostic groups and outcomes

**DOI:** 10.1038/s41408-021-00538-9

**Published:** 2021-09-21

**Authors:** Zaid H. Abdel-Rahman, Michael G. Heckman, Theodora Anagnostou, Launia J. White, Sara M. Kloft‐Nelson, Ryan A. Knudson, Hassan B. Alkhateeb, Lisa Z. Sproat, Nandita Khera, Hemant S. Murthy, Ernesto Ayala, William J. Hogan, Vivek Roy, Jess F. Peterson, Mohamed A. Kharfan-Dabaja, Rhett P. Ketterling, Mark R. Litzow, Linda B. Baughn, Mrinal Patnaik, Patricia T. Greipp, James M. Foran

**Affiliations:** 1 Division of Hematology and Medical Oncology, Mayo Clinic, Jacksonville, FL USA; 2 Division of Biomedical Statistics and Informatics, Mayo Clinic, Jacksonville, FL USA; 3 Bone Marrow Transplantation Service, Department of Medicine, Memorial Sloan Kettering Cancer Center, New York, NY USA; 4 Cytogenetics Core Laboratory, Medical Genome Facility, Mayo Clinic, Rochester, MN USA; 5 Division of Hematology, Mayo Clinic, Rochester, MN USA; 6 Division of Hematology and Medical Oncology, Mayo Clinic, Phoenix, AZ USA; 7 Division of Laboratory Genetics and Genomics, Mayo Clinic, Rochester, MN USA

**Keywords:** Acute lymphocytic leukaemia, Cytogenetics

Dear Editor,

Philadelphia-like acute lymphoblastic leukemia (Ph-like ALL) is a high-risk subset of B-cell ALL that shares a similar gene expression profile with *BCR-ABL1*-positive ALL but lacks the *BCR-ABL1* gene fusion [1, 2]. This subgroup was first described in children and has been associated with a poor prognosis in both children and adults [1–3], however, data regarding optimal management and the specific role of allogeneic transplantation (allo-HCT) in adults is limited [4, 5].

More than 90% of patients with Ph*-*like ALL have distinct genomic alterations which are broadly divided into ABL-class fusions (*ABL1*, *ABL2*, *CSF1R*, or *PDGFRB*) and alterations activating the *JAK-STAT* pathway (*CRLF2*, *JAK2*, and *EPOR*) [6–8]. The Mayo Clinic Clinical Genomics Laboratory developed a tiered diagnostic approach that employs a targeted FISH panel [9] to identify Ph-like ALL-specific gene fusions, which allows for timely recognition of this entity and provides information that would otherwise be unobtainable in a setting where gene expression profiling or RNA-based or whole transcriptome sequencing is not available.

In this study, we identify patients with Ph-like ALL by applying our targeted FISH panels on remnant diagnostic leukemia cytogenetic pellets from patients identified and treated at the Mayo Clinic Cancer Center (01/01/2008–12/31/2019) and examine clinical outcomes with a focus on the role of induction therapy, minimal residual disease (MRD) and allo-HCT in comparison to other cytogenetic groups. Continuous variables were summarized using the sample median and range. Categorical variables were summarized with the number and percentage of patients. Comparisons of characteristics between Ph-like, Ph-pos, and Ph-neg patients were made using a Kruskal–Wallis rank-sum test (continuous variables) or Fisher’s exact test (categorical variables). Survival after ALL diagnosis and after allo-HCT was estimated using the Kaplan-Meier method. Associations of variables with survival were evaluated using unadjusted and multivariable Cox proportional hazards regression models. Statistical analyses were performed using SAS (version 9.4; SAS Institute, Inc., Cary, North Carolina). This study was approved by the Mayo Clinic Institutional Review Board.

We identified 365 patients with ALL during the study period, median follow-up after ALL diagnosis was 30.8 months (range: 0.7–216.3 months). The median age was 48 years (range: 18–88 years). Patient characteristics for the overall cohort are summarized in Table 1.

**Table 1:** Patients characteristics.

Variable	All patients (*N* = 365)	Ph-like ALL (*N* = 33)	Ph-pos ALL (*N* = 132)	Ph-neg ALL (*N* = 200)	*p*-Value
Age at diagnosis (years)	48 (17, 88)	39 (17, 88)	50 (21, 79)	49 (17, 83)	**0.01**
Age ≤ 40 years	133 (36.3%)	17 (51.5%)	37 (28.0%)	79 (39.5%)	**0.02**
Sex (male)	198 (54.2%)	22 (66.7%)	72 (54.5%)	104 (52.0%)	0.30
Race (Caucasian)	311 (87.4%)	28 (90.3%)	113 (87.6%)	170 (86.7%)	0.91
Ethnicity (Hispanic/Latino)	49 (14.6%)	9 (28.1%)	14 (11.7%)	26 (14.1%)	0.07
WBC	9.0 (0.0, 700.0)	27.9 (0.6, 199.0)	21.5 (0.8, 571.0)	4.5 (0.0, 700.0)	**<0.001**
Hb	9.1 (0.0, 82.0)	9.3 (6.1, 15.6)	9.7 (4.0, 16.4)	9.0 (0.0, 82.0)	0.22
Platelets	49 (0, 519)	35 (3, 237)	44 (2, 351)	54 (0, 519)	0.33
CNS involvement at diagnosis	39 (10.7%)	4 (12.1%)	19 (14.4%)	16 (8.0%)	0.17
*Induction regimen*					**0.002**
Hyper CVAD	238 (65.2%)	16 (48.5%)	99 (75.0%)	123 (61.5%)	
Pediatric regimens	60 (16.4%)	11 (33.3%)	9 (6.8%)	40 (20.0%)	
ECOG regimens	40 (11.0%)	4 (12.1%)	13 (9.8%)	23 (11.5%)	
Others	27 (7.4%)	2 (6.1%)	11 (8.3%)	14 (7.0%)	
Allo-HCT	253 (69.3%)	18 (54.5%)	109 (82.6%)	126 (63.0%)	**<0.001**

The sample median (minimum, maximum) is given for continuous variables. For the overall tests of difference, *p*-values result from Fisher’s exact test (categorical variables) or a Kruskal–Wallis rank-sum test (continuous variables). Statistically significant findings are in bold.

To identify patients with Ph-like ALL, we excluded cytogenetic groups known not to harbor a Ph-like genetic signature (*BCR/ABL1* fusion, *KMT2A* rearrangements, *TCF3–PBX1* fusion, and *ETV6/RUNX1* fusion) and we applied the FISH panel on all remaining diagnostic cytogenetic pellets (*N* = 128). Of the 365 patients, 33 (9%) patients with Ph-like ALL were identified; of these, 22 (67%) had *CRLF2* rearrangements, 5 (12%) had *JAK-STAT* pathway alterations or *EPOR* rearrangements, and 6 (18%) patients had *ABL*-class fusions (Supplementary Table S1). Of the remaining patients, 132 (36%) had Ph-pos ALL and 200 (55%) had Ph-neg ALL. All patients with Ph-pos ALL received *BCR*/*ABL1* tyrosine kinase inhibitors as part of their induction regimen (dasatinib; *n* = 86, imatinib; *n* = 44, ponatinib; *n* = 2) based on physician choice. Five patients with Ph-like ALL received targeted therapies as part of their induction regimen; imatinib, *n* = 1 for *PDGFRB* rearrangement; dasatinib, *n* = 2 for a *RANBP2*/*ABL1* fusion and an *ABL2/RCSD1* fusion; ruxolitinib, *n* = 1 for *IGH/CRLF2* fusion and ponatinib, *n* = 1 for *NUP214*/*ABL1* fusion.

Patients with Ph-like ALL were significantly younger, with a median age of 39 years (compared with 50 years for patients with Ph-pos and 49 years for Ph-neg ALL; *P* = 0.01) and more likely to be of Hispanic ethnicity (28.1%, vs. 11.7% for Ph-pos and 14.1% for Ph-neg, *P* = 0.07). Patients with Ph-like ALL presented with higher WBC (median 27.9 × 10^9^/L, compared with 21.5 × 10^9^/L for Ph-pos and 4.5 × 10^9^/L for Ph-neg ALL; *P* < 0.001), were more likely to have received pediatric regimens (33.3%, compared with 6.8% for Ph-pos and 20.0% for Ph-neg ALL; *P* = 0.002) and less likely to proceed to allo-HCT (54.5%, compared with 82.6% for Ph-pos and 63.0% for Ph-neg, *P* < 0.001).

Patients with Ph-like ALL were significantly less likely to achieve CR (91% vs. 99% vs. 95%, *P* = 0.02), and more likely to be MRD positive (64% vs. 34% vs. 36%, *P* = 0.03), measured at the end of induction using a flow cytometry assay with a sensitivity of 0.01%. They experienced a higher relapse rate (5-year cumulative incidence: 39% vs. 24% vs. 37%, *P* = 0.02 for overall comparison, *P* = 0.93 for Ph-like vs. Ph-neg ALL) and lower OS (5-year estimates: 41% vs. 64% vs. 49%, *P* = 0.02 for overall comparison, *P* = 0.87 for Ph-like vs. Ph-neg ALL, Fig. 1A).

**Fig. 1:**
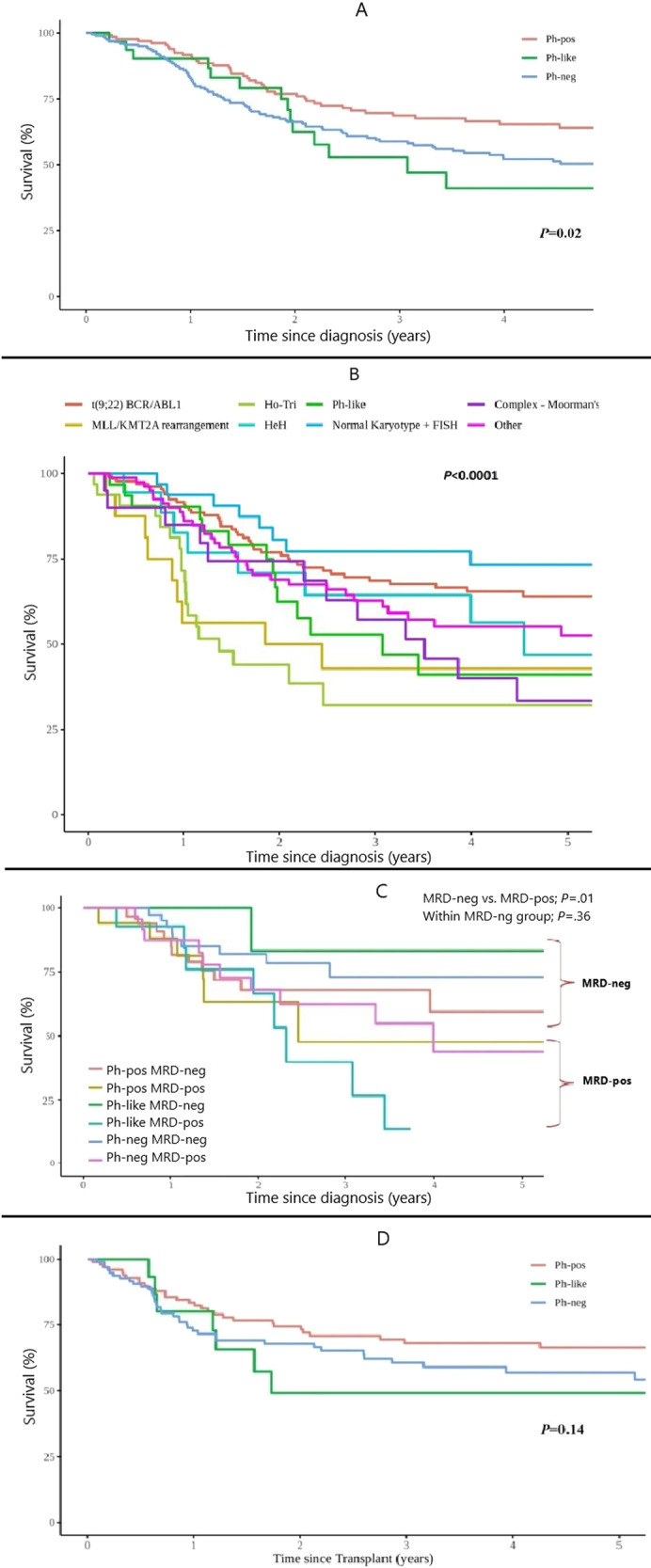
Outcomes of patients with Ph-like ALL in comparison to other groups. **A** Overall survival of Ph-like, Ph-pos, and Ph-neg ALL. **B** Overall survival by cytogenetic subgroup. **C** Overall survival by MRD status. **D** Overall survival after allo-HCT.

Interestingly, we observed that patients with Ph-like ALL typically experienced relapse later during their treatment course than other groups, with approximately 74% of relapses occurring between 12 and 36 months, compared to only 33 and 46% of relapses occurring during the same time period in Ph-pos and Ph-neg ALL, respectively. Reasons for this are uncertain, but it may relate to the use of continuation/maintenance therapy on pediatric regimens which were more commonly employed in this group.

MRD negativity after induction was associated with significantly better OS (Fig. 1C). There was no statistically significant difference in OS between the three major groups among patients who did achieve MRD negativity (i.e., Ph-pos vs. Ph-like vs. Ph-neg, *P* = 0.36).

In a multivariable analysis for overall survival (Supplementary Table S2), independent associations were observed for age at ALL diagnosis (HR [per 10-year increment]: 1.26, 95% CI: 1.12–1.41, *P* < 0.001) and Ph-like status identified by the FISH algorithm (Ph-pos vs. Ph-neg, HR = 0.54, *P* < 0.001; Ph-like vs. Ph-neg, HR = 1.28, *P* = 0.40, overall *P* = 0.001). The use of pediatric-inspired regimens was also independently associated with improved OS in comparison to hyper-CVAD (HR = 0.56, 95% CI: 0.32–0.98, *P* = 0.048).

Of the 365 patients, 252 underwent allo-HCT at some point in their disease course, but only patients who underwent allo-HCT in CR1 were included in this subgroup analysis (*N* = 212), and their characteristics are shown in Supplementary Table S3. Median follow-up after allo-HCT was 28.1 months (range: 0.5–146.2 months). There were no statistically significant differences in outcomes between the three major groups (Ph-like, Ph-pos, and Ph-neg ALL) after allo-HCT (Fig. 1D and Supplementary Tables S4 and S5). However, patients who achieved MRD negativity after induction had improved OS after allo-HCT (Supplementary Fig. 1, MRD− vs. MRD+, *P* = 0.02).

In this study, we report clinical outcomes from the Mayo Clinic adult B-ALL cohort, incorporating an accessible and practical FISH-based diagnostic platform for Ph-like ALL, with emphasis on induction regimen, achievement of MRD negativity, and allo-HCT. In comparison to Ph-pos and Ph-neg ALL, our findings show that patients with Ph-like ALL present at a younger age and with a higher WBC count. They were also less likely to achieve CR and MRD negativity after induction, more likely to relapse (particularly at a later time point), and have an inferior OS.

Patients with Ph-like ALL who remained MRD positive after frontline induction therapy had an exceptionally low OS compared to those who achieved MRD negativity, highlighting the significance of achieving early MRD negativity in this subgroup. Additionally, there was no difference in overall survival in patients who achieved MRD negativity in the three groups, confirming the important prognostic significance of MRD in ALL that often surpasses that of cytogenetic risk groups [10–12].

Even though these patients were younger and more likely to be treated with pediatric-inspired regimens, two factors associated with better outcomes in our cohort on multivariable analysis, the 5-year survival of patients with Ph-like ALL in our cohort was significantly lower than most cytogenetic groups.

The adoption of *BCR/ABL1-*directed TKI therapy for Ph-pos ALL, together with allogeneic transplant in CR1, has had a significant impact on the outcome on what was previously considered a high-risk group. Along similar lines, this paves the road for future studies on Ph-like ALL (as another kinase-driven ALL) to evaluate if a similar impact can be achieved in patients with this entity, with the choice of TKI dictated by specific targetable genetic alterations.

In the allo-HCT cohort, there were no statistically significant differences in outcomes between the three groups, indicating the importance of allo-HCT in this setting and supporting its role in the management of patients with Ph-like ALL.

We acknowledge several limitations of our study: the first being the retrospective design of the study which introduces biases into the data collection. The second limitation being the relatively small sample sizes of some of the groups that were examined, therefore, the possibility of a type II error is important to consider. Because the diagnostic algorithm was not available at the time of diagnosis for most patients, some cases of Ph-like ALL were not recognized in practice, and this in turn impacted selection and treatment strategies. Future studies employing the fluorescence in situ hybridization (FISH) diagnostic strategy prospectively together with RNA sequencing will help confirm its immediate availability and utility in practice.

With a 5-year OS of only 41%, Ph-like is considered one of the highest-risk cytogenetic groups in adult ALL. FISH is a practical and available tool to help identify this entity in practice. The dismal outcome with standard therapy calls for the coordinated exploration of the role of targeted therapies, based on kinase-specific drivers in a similar fashion to Philadelphia-positive ALL, as part of the induction, consolidation, and maintenance phases of treatment in order to improve clinical outcomes for adults with this disease.

## Supplementary information

Supplemental Material

## Data Availability

For original data, please contact Foran.James@mayo.edu.
