# Supplementary material for: Identification of adult Philadelphia-like acute lymphoblastic leukemia using a FISH‐based algorithm distinguishes prognostic groups and outcomes
Source: Blood Cancer J. 2021 Sep 21;11(9):156. doi: 10.1038/s41408-021-00538-9 (PMC8455651; doi:10.1038/s41408-021-00538-9)

**Supplementary Material**

**Identification of adult Philadelphia-like acute lymphoblastic leukemia using a FISH‐based algorithm distinguishes prognostic groups and outcomes**

Zaid H. Abdel-Rahman, MD, Michael G. Heckman, MS, Theodora Anagnostou, MD, Launia J. White, Sara M. Kloft‐Nelson, Ryan A. Knudson, Hassan B Alkhateeb, MD, Lisa Z. Sproat, MD, Nandita Khera, MD, MPH, Hemant S. Murthy, MD, Ernesto Ayala, MD, William J. Hogan, MB, BCh, Vivek Roy, MD, Jess F. Peterson, MD, Mohamed A. Kharfan-Dabaja, MD, MBA, Rhett P. Ketterling, MD, Mark R. Litzow, MD, Linda B. Baughn, PhD, Mrinal Patnaik, MD, Patricia T. Greipp, DO* and James M. Foran, MD*

* Drs James Foran and Patricia Greipp contributed to this manuscript equally.

**Supplementary Table S1: Cytogenetic alterations in Ph-like cases in the Mayo Clinic ALL Cohort**

| Ph-like group | N | Rearrangements | Number of patients with rearrangement |
| --- | --- | --- | --- |
| *CRLF2* rearrangement | 22 | *CRLF2/IGH* | 16 |
|  |  | *CRLF2/P2RY8* | 4 |
|  |  | Unidentified partners | 2 |
| *JAK-STAT* alterations | 5 | *JAK2* rearrangements/mutation | 4 |
|  |  | *EPOR* rearrangement | 1 |
| *ABL*-class | 6 | *PDGFRB* rearrangement | 2 |
|  |  | *NUP214/ABL1* | 2 |
|  |  | *ABL1/RANBP2* | 1 |
|  |  | *ABL2/RCSD1* | 1 |

**Supplementary Table S2: Associations of patient characteristics with survival after ALL diagnosis**

|  | Unadjusted analysis | | Multivariable analysis | |
| --- | --- | --- | --- | --- |
| Variable | HR (95% CI) | P-value | HR (95% CI) | P-value |
| Age (10-year increase) | **1.27 (1.14, 1.41)** | **<0.001** | **1.25 (1.12, 1.40)** | **<0.001** |
| Sex (Male) | 1.03 (0.75, 1.42) | 0.86 | 1.07 (0.78, 1.48) | 0.67 |
| Ethnicity (Hispanic/Latino) | 1.13 (0.70, 1.82) | 0.62 | 1.28 (0.79, 2.08) | 0.31 |
| Race (non-Caucasian) | 1.06 (0.65, 1.74) | 0.81 | 1.11 (0.68, 1.82) | 0.68 |
| WBC (1 unit increase on cube root scale) | 0.96 (0.85, 1.08) | 0.46 | 1.10 (0.97, 1.25) | 0.13 |
| Hb (1-unit increase) | 1.02 (0.99, 1.05) | 0.19 | 1.01 (0.98, 1.05) | 0.44 |
| Platelets (1 unit increase on cube root scale) | 0.95 (0.82, 1.09) | 0.44 | 0.93 (0.81, 1.07) | 0.31 |
| Ph-like status | **Overall test of difference: P=0.02** | | **Overall test of difference: P=0.001** | |
| Ph-neg | 1.00 (reference) | N/A | 1.00 (reference) | N/A |
| Ph-pos | **0.62 (0.43, 0.88)** | **0.008** | **0.53 (0.37, 0.77)** | **<0.001** |
| Ph-like | 1.05 (0.60, 1.84) | 0.87 | 1.27 (0.72, 2.24) | 0.41 |
| CNS involvement | 0.81 (0.47, 1.41) | 0.46 | 0.81 (0.46, 1.40) | 0.45 |
| Induction regimen | **Overall test of difference: P=0.04** | | Overall test of difference: P=0.25 | |
| HyperCVAD | 1.00 (reference) | N/A | 1.00 (reference) | N/A |
| Pediatric regimens | **0.47 (0.28, 0.80)** | **0.005** | **0.57 (0.33, 1.00)** | **0.048** |
| ECOG regimens | 1.00 (0.61, 1.66) | 0.99 | 0.87 (0.53, 1.43) | 0.58 |
| Others | 0.92 (0.51, 1.68) | 0.79 | 0.87 (0.48, 1.58) | 0.64 |
| HR=hazard ratio; CI=confidence interval. HRs, 95% CIs, and p-values result from Cox proportional hazards regression models. HRs correspond to presence of the given characteristic (categorical variables) or the increase given in parenthesis (continuous variables). Multivariable models were adjusted for age, Ph-like status, and induction regimen. | | | | |

**Supplementary Table S3: Comparisons of characteristics between Ph-like, Ph+, and Ph- patients in the allo-HCT cohort**

| Variable | N | All  (N=212) | Ph-like (N=16) | Ph+  (N=99) | Ph-  (N=97) | P-value |
| --- | --- | --- | --- | --- | --- | --- |
| Age at diagnosis (years) | 212 | 49 (17, 70) | 47 (17, 61) | 48 (21, 70) | 52 (19, 70) | 0.45 |
| Sex (Male) | 212 | 104 (49.1%) | 8 (50.0%) | 54 (54.5%) | 42 (43.3%) | 0.28 |
| Race (Caucasian) | 208 | 182 (87.5%) | 15 (100.0%) | 88 (89.8%) | 79 (83.2%) | 0.12 |
| Ethnicity (Hispanic/Latino) | 198 | 31 (15.7%) | 3 (18.8%) | 10 (11.0%) | 18 (19.8%) | 0.27 |
| WBC | 190 | 10.2 (0, 571) | 30.5 (0.6, 199) | 24.8 (1.2, 571) | 5.0 (0.0, 312) | **<0.001** |
| Hb | 170 | 9.1 (0.0, 16.6) | 9.4 (6.5, 15.6) | 9.6 (4.0, 16.4) | 8.9 (0.0, 16.6) | 0.57 |
| Platelets | 176 | 51 (0, 519) | 46 (6, 237) | 43 (2, 259) | 57 (0, 519) | 0.83 |
| CNS involvement | 212 | 25 (11.8%) | 1 (6.3%) | 13 (13.1%) | 11 (11.3%) | 0.85 |
| Induction regimen | 212 |  |  |  |  | 0.14 |
| HyperCVAD |  | 145 (68.4%) | 9 (56.3%) | 76 (76.8%) | 60 (61.9%) |  |
| Pediatric regimens |  | 28 (13.2%) | 4 (25.0%) | 7 (7.1%) | 17 (17.5%) |  |
| ECOG regimens |  | 22 (10.4%) | 2 (12.5%) | 9 (9.1%) | 11 (11.3%) |  |
| Others |  | 17 (8.0%) | 1 (6.3%) | 7 (7.1%) | 9 (9.3%) |  |
| MRD (positive) | 87 | 33 (37.9%) | 7 (53.8%) | 13 (37.1%) | 13 (33.3%) | 0.42 |
| Conditioning regimen | 212 |  |  |  |  | 0.056 |
| MAC |  | 160 (75.5%) | 12 (75.0%) | 82 (82.8%) | 66 (68.0%) |  |
| NMA/RIC |  | 52 (24.5%) | 4 (25.0%) | 17 (17.2%) | 31 (32.0%) |  |
| Donor type | 212 |  |  |  |  | 0.17 |
| Matched related |  | 78 (36.8%) | 7 (43.8%) | 29 (29.3%) | 42 (43.3%) |  |
| Haploidentical |  | 19 (9.0%) | 0 (0.0%) | 9 (9.1%) | 10 (10.3%) |  |
| Matched unrelated |  | 115 (54.2%) | 9 (56.3%) | 61 (61.6%) | 45 (46.4%) |  |
| The sample median (minimum, maximum) is given for continuous variables. P-values result from Fisher’s exact test (categorical variables) or a Kruskal-Wallis rank sum test (continuous variables). | | | | | | |

**Supplementary Table S4: Comparisons of allo-HCT outcomes between Ph-like, Ph-pos, and Ph-neg ALL**

|  |  | Cumulative incidence (%) (95% CI) | | | |
| --- | --- | --- | --- | --- | --- |
| Variable | N | Ph-like ALL (N=16) | Ph-pos ALL (N=99) | Ph-neg ALL (N=97) | P-value |
| Overall Survival | 212 |  | | | |
| 1-year after transplant |  | 80.0 (61.5, 100) | 83.4 (76.3, 91.2) | 72.6 (64.0, 82.4) | 0.14 |
| 3-years after transplant |  | 49.1 (23.7, 85.0) | 68.0 (58.9, 78.4) | 60.6 (50.6, 72.1) |  |
| 5-years after transplant |  | 49.1 (21.2, 85.0) | 66.2 (56.7, 77.1) | 56.8 (45.9, 69.1) |  |
| Relapse | 212 |  | | | |
| 1-year after transplant |  | 13.3 (2.0, 35.6) | 10.3 (5.2, 17.3) | 10.8 (5.5, 18.1) | 0.38 |
| 3-years after transplant |  | 27.6 (7.7, 52.4) | 14.0 (7.8, 21.9) | 17.8 (10.3, 26.9) |  |
| 5-years after transplant |  | 27.6 (7.7, 52.4) | 14.0 (7.8, 21.9) | 20.0 (11.7, 29.9) |  |
| Non-relapse mortality | 212 |  | | | |
| 1-year after transplant |  | 20.0 (4.5, 43.3) | 12.4 (6.8, 19.9) | 20.8 (13.1, 29.7) | 0.54 |
| 3-years after transplant |  | 26.7 (7.7, 50.7) | 19.4 (12.0, 28.2) | 24.9 (16.3, 34.4) |  |
| 5-years after transplant |  | 26.7 (7.7, 50.7) | 21.2 (13.2, 30.4) | 28.8 (19.1, 39.2) |  |
| P-values result from a log-rank test. | | | | | |

**Supplementary Table S5: Associations of patient characteristics with survival after allo-HCT**

|  | Unadjusted analysis | | Multivariable analysis | |
| --- | --- | --- | --- | --- |
| Variable | HR (95% CI) | P-value | HR (95% CI) | P-value |
| Age (10-year increase) | **1.32 (1.08, 1.62)** | **0.007** | 1.17 (0.93, 1.48) | 0.18 |
| Sex (Male) | 1.00 (0.64, 1.57) | 0.99 | 1.07 (0.68, 1.68) | 0.77 |
| Ethnicity (Hispanic/Latino) | 1.08 (0.58, 2.02) | 0.80 | 1.17 (0.62, 2.21) | 0.62 |
| Race (non-Caucasian) | 0.80 (0.38, 1.67) | 0.55 | 0.94 (0.44, 1.99) | 0.86 |
| WBC (1 unit increase on cube root scale) | 0.89 (0.76, 1.04) | 0.14 | 0.95 (0.80 1.12) | 0.52 |
| Hb (1 unit increase) | 0.97 (0.89, 1.05) | 0.41 | 0.95 (0.87, 1.04) | 0.29 |
| Platelets (1 unit increase on cube root scale) | 0.94 (0.79, 1.12) | 0.50 | 0.92 (0.77, 1.11) | 0.39 |
| Ph-like status | Overall test of difference: P=0.15 | | Overall test of difference: P=0.44 | |
| Ph- | 1.00 (reference) | N/A | 1.00 (reference) | N/A |
| Ph+ | 0.65 (0.41, 1.05) | 0.076 | 0.78 (0.48, 1.26) | 0.31 |
| Ph-like | 1.16 (0.52, 2.59) | 0.72 | 1.24 (0.55, 2.79) | 0.60 |
| CNS involvement | 0.88 (0.42, 1.82) | 0.72 | 1.00 (0.48, 2.08) | 0.99 |
| Induction regimen | Overall test of difference: P=0.37 | | Overall test of difference: P=0.38 | |
| HyperCVAD | 1.00 (reference) | N/A | 1.00 (reference) | N/A |
| Pediatric regimens | 0.64 (0.30, 1.34) | 0.23 | 0.95 (0.42, 2.18) | 0.91 |
| ECOG regimens | 1.28 (0.65, 2.51) | 0.48 | 1.54 (0.77, 3.10) | 0.22 |
| Others | 0.63 (0.25, 1.58) | 0.33 | 0.58 (0.23, 1.46) | 0.25 |
| Conditioning regimen |  |  |  |  |
| MAC | 1.00 (reference) | N/A | 1.00 (reference) | N/A |
| NMA/RIC | **2.05 (1.26, 3.32)** | **0.004** | 1.65 (0.93, 2.95) | 0.089 |
| Donor type | **Overall test of difference: P=0.008** | | **Overall test of difference: P=0.011** | |
| Matched related | 1.00 (reference) | N/A | 1.00 (reference) | N/A |
| Haploidentical | 0.58 (0.23, 1.47) | 0.25 | 0.52 (0.21, 1.33) | 0.17 |
| Matched unrelated | **0.49 (0.31, 0.77)** | **0.002** | **0.50 (0.32, 0.80)** | **0.004** |
| HR=hazard ratio; CI=confidence interval. HRs, 95% CIs, and p-values result from Cox proportional hazards regression models. HRs correspond to presence of the given characteristic (categorical variables) or the increase given in parenthesis (continuous variables). Multivariable models were adjusted for age, conditioning regimen, and donor type. | | | | |

**Supplementary Figure 1: Overall survival after allo-HCT by MRD status in Ph-like, Ph-pos, and Ph-neg** **ALL patients**


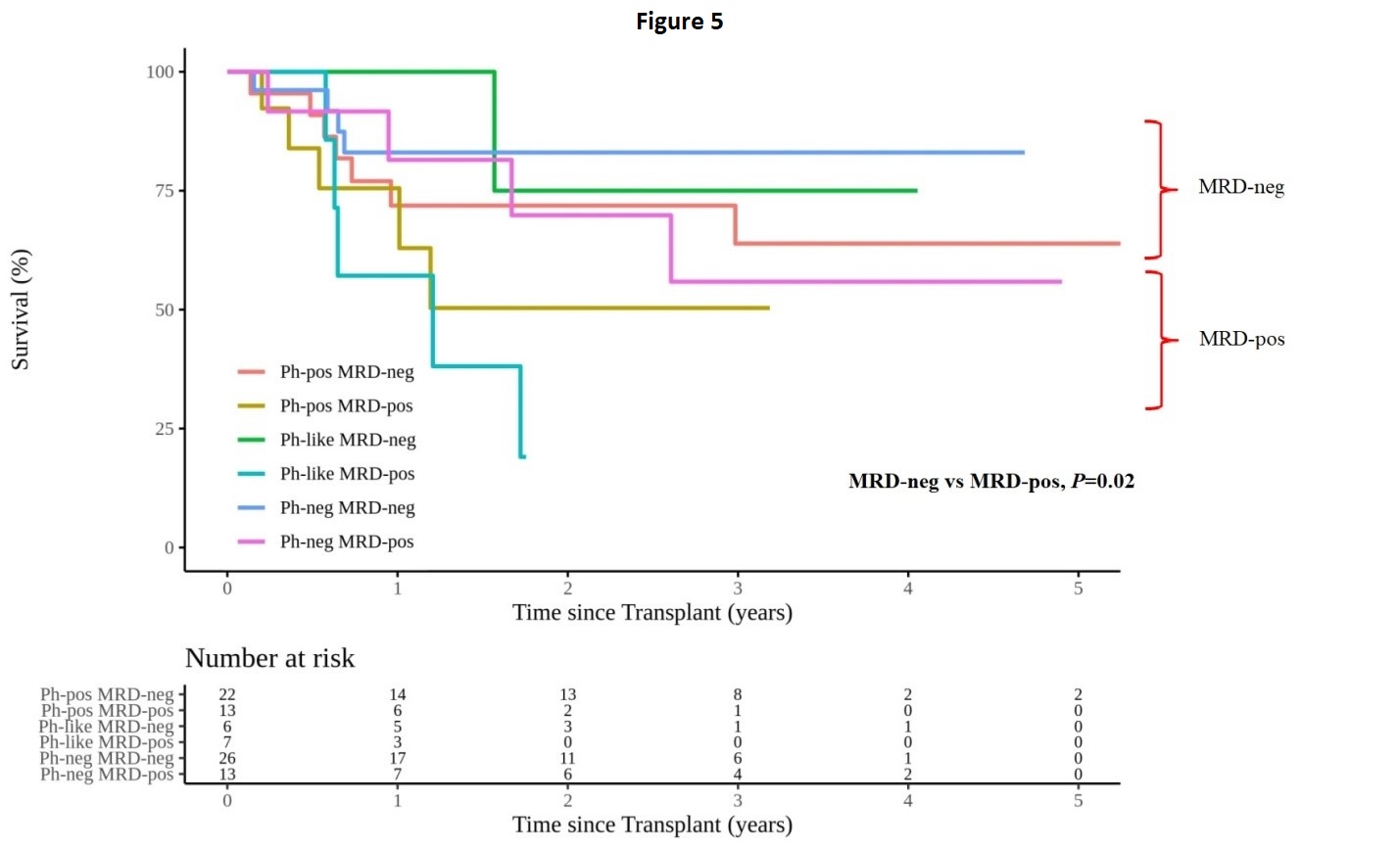

Supplement: Supplementary file 1 — Supplemental Material [file 41408_2021_538_MOESM1_ESM.docx]
